# Predictors of pathologic complete response to neoadjuvant treatment in HER2-overexpressing breast cancer: a retrospective analysis using real-world data

**DOI:** 10.3332/ecancer.2022.1338

**Published:** 2022-01-06

**Authors:** Isabel Saffie Vega, Jorge Sapunar Zenteno, Felipe Buscaglia Fernandez, Felipe Reyes Cosmelli, Rodrigo Lagos Chavez, Badir Chahuán Manzur

**Affiliations:** 1 Breast Oncologic and Reconstructive Surgery Unit, Instituto Oncológico Fundación Arturo López Pérez, Santiago 7500691, Chile; 2 Epidemiologic and Clinical Research Unit, Department of Cancer Research, Instituto Oncológico Fundación Arturo López Pérez, Santiago 7500691, Chile; 3 Department of Internal Medicine, School of Medicine, Universidad de La Frontera, Temuco 4811230, Chile; 4 Pathology Anatomy Unit, Instituto Oncológico Fundación Arturo López Pérez, Santiago 7500691, Chile; 5 Medical Oncology Unit, Instituto Oncológico Fundación Arturo López Pérez, Santiago 7500691, Chile

**Keywords:** breast cancer, HER2 positive, neoadjuvant chemotherapy, pathologic complete response, trastuzumab, pertuzumab

## Abstract

The human epidermal growth factor receptor 2 (ERBB2, HER2 or HER2/neu) is a transmembrane tyrosine kinase receptor that is overexpressed in approximately 20% of breast cancers. The use of the anti-HER2 monoclonal antibodies Pertuzumab and Trastuzumab in association with chemotherapy has achieved a higher percentage of pathologic complete response (pCR) than conventional chemotherapy. The purpose of our study was to identify factors that could affect the therapeutic response of patients with breast cancer and HER2 overexpression treated with cytotoxic chemotherapy plus double HER2 blockade in neoadjuvant setting at Fundación Arturo López Pérez (FALP). A case–control study was designed to evaluate the effect of clinical and histopathological variables on the response to neoadjuvant therapy. Ninety-four women with non-metastatic breast cancer and HER2 overexpression received neoadjuvant combination chemotherapy with Trastuzumab and Pertuzumab at FALP during the period 2017–2020. Seventy percent of patients achieved pCR, and in the group of hormone receptor negative patients, 89% of patients achieved pCR. Different variables were analysed trying to look for clinicopathological predictors of complete response. This study provides us with real-world data on the efficacy of using this treatment combination in our population of HER2-overexpressing breast cancer patients.

## Introduction

Breast cancer is the most commonly diagnosed malignancy, regardless of the level of development of regions and countries [1]. One in eight women will have breast cancer in her lifetime and it is the leading cause of cancer mortality in the Chilean population with an age-standardised rate of 10.2 per 100,000 women [2]. The World Health Organization considers it one of the main public health problems in developed and developing countries.

Today, breast cancer is classified into different molecular subtypes that correlate closely with the presence or absence of immunohistochemical markers: hormone receptor positive/HER2 negative, HER2 positive and triple negative in which case there is no expression of hormone receptors or HER2 protein. Each corresponds to approximately 70%, 20% and 10% of breast cancers, respectively. These breast cancer subtypes have different risk profiles and treatment strategies [3].

Human epidermal growth factor receptor 2 (ERBB2, HER2 or HER2/neu) is a molecular target consisting of a transmembrane tyrosine kinase-like receptor. This epidermal growth factor is overexpressed or amplified in approximately 20% of breast cancers and has been associated with worse prognosis in the absence of targeted systemic therapy [4]. HER2-overexpressing tumours benefit from anti-HER2 target therapies such as the monoclonal antibodies Trastuzumab and Pertuzumab, as well as small molecule tyrosine kinase inhibitors such as Lapatinib and Neratinib among others. These treatments have prolonged progression-free survival and overall survival in the subgroup of patients with metastatic breast cancer and also improved pathologic complete response (pCR) rates in patients with less advanced stages receiving neoadjuvant therapy [5].

The clinical benefit of combining Trastuzumab and Pertuzumab with chemotherapy was initially demonstrated in the CLEOPATRA study where there was a significant increase in progression-free survival and overall survival in patients with HER2-overexpressing tumours and metastatic disease [8, 9].

Based on these results, the use of Pertuzumab, Trastuzumab and chemotherapy was evaluated as neoadjuvant therapy in patients with HER2 disease in two phase 2 studies [6, 7]. In these studies, a higher percentage of patients achieved pCR with combined anti-HER2 treatment added to conventional chemotherapy.

The results of combined anti-HER2 treatment in HER2-overexpressing breast cancers in both metastatic and neoadjuvant settings have not been reported in Chile.

The aim of our study was to assess the effect of combined anti-HER2 treatment in neoadjuvant setting and to identify factors that could affect the therapeutic response of patients diagnosed with breast cancer and HER2 overexpression treated at the Instituto Oncológico Fundación Arturo López Pérez (FALP).

## Method

A case–control study was designed to evaluate the effect of clinical and histopathological variables on the response to combined neoadjuvant therapy with Trastuzumab and Pertuzumab associated with cytotoxic chemotherapy in women with breast cancer and HER2 overexpression attended at FALP between the years 2017 and 2020.

All women with diagnosis of breast cancer and HER2 overexpression confirmed by the fluorescence *in situ* hybridization (FISH) technique, without metastatic disease and who underwent combined anti-HER2 therapy in neoadjuvant setting in our institution between the mentioned years were included in the study. All patients also received cytotoxic chemotherapy.

***Cases*** were those women who achieved pCR (ypT0/isN0) at surgery after neoadjuvant chemotherapy (NACT) and ***controls*** were those who did not.

The exposure variables evaluated were age, body mass index (BMI), duration and type of neoadjuvant therapy, staging, T and N (cTNM) category [10], maximum tumour size at diagnosis and axillary involvement by ultrasonography. Histopathology of the *core* biopsy considered the molecular profile (pure HER2 versus HER2/Luminal), oestrogen receptor (ER) and progesterone receptor (PR) expression, Ki67 index (dichotomised as > or <20%), HER2 copy number and HER2/centromere ratio reported by FISH study. Every patient with HER2 2+ or 3+ was confirmed by FISH.

The response variable was whether or not a complete pathologic response was achieved at surgery after combined neoadjuvant therapy.

Statistical analysis was performed with R software version 4.0.5 (R Core Team, 2018. Vienna, Austria). The effect of each exposure variable on pathologic response was evaluated using the Wilcoxon sign test for continuous variables, Fisher’s exact test for nominal variables and the Cochran–Armitage test for ordinal variables. The combined effect of the exposure variables was then evaluated by multiple logistic regression and the StepWise algorithm was applied to determine which variables best predict the complete pathological response for inclusion in the final model. A model with dichotomous molecular profiling (pure HER2 versus HER2/Luminal) and a second model with molecular profiling broken down into pure HER2, HER2/Luminal ER (+) and HER2/Luminal ER (+) and PR (+) were generated. The magnitude of the effect of the exposure variables on the response variable is presented with Odds Ratio (OR).

Finally, considering that only in 39 patients we were able to obtain the HER2 copy number and HER2/centromere ratio described in the FISH study, the association between this variable and pathologic response was analysed univariate.

## Results

**Figure 1.**
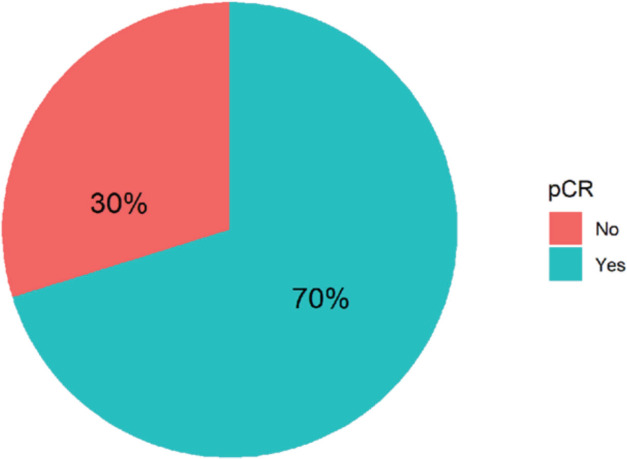
Types of pathological response in 94 women with breast cancer and HER-2 overexpression treated with NACT plus double anti-HER2 blockade at FALP in the period 2017–2020.

Ninety-four women with non-metastatic breast cancer and HER2 overexpression received neoadjuvant combination chemotherapy with Trastuzumab and Pertuzumab at FALP during the period 2017–2020. Figure 1 shows that 70% of all patients achieved complete pathologic response. Table 1 shows the demographic, clinical, and anatomopathological characteristics of the group analysed.

Table 2 compares demographic, clinical, and histopathologic variables of patients with complete pathologic response (cases) and those without complete pathologic response (controls). We highlight a higher proportion of women with cancer in more advanced stages and with Ki67 > 20% when pCR was achieved.

When developing the first multiple logistic regression model and applying the StepWise algorithm (Table 3), it was found that the OR of not having a complete pathological response in women with HER2/Luminal breast cancer was 8.15 with respect to the pure HER2 profile (95% CI: 2.43–37.7), while the OR for clinical stage change (I, II, III) was 0.28 (95% CI: 0.06–1.05).

When developing the multiple logistic regression model with the molecular profile broken down into pure HER2 (base level), HER2/Luminal with ER (+) and HER2/Luminal ER (+) and PR (+) and applying the StepWise algorithm (Table 3), it was found that the latter profile with expression of both receptors had an OR of not having a complete pathological response of 10.42 (95% CI: 3.03–47.9).

**Figure 2.**
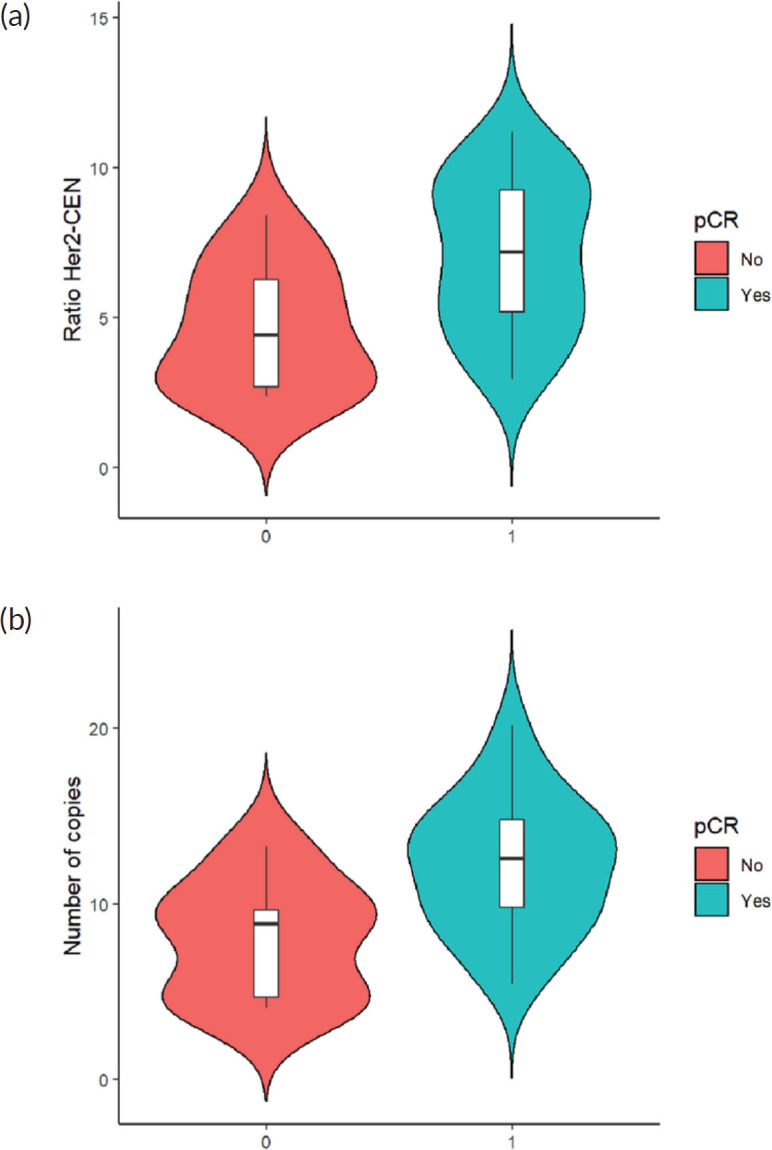
(a): HER 2/centromere ratio value on FISH of core biopsy of women with breast cancer and HER 2 overexpression, who received combined neoadjuvant therapy at FALP in the period 2017–2020, according to pathological response at surgery. Both groups were compared by Wilcoxon sign test, resulting in a statistic *W* = −2.84 and a *p*-value = 0.0037. (b): HER2 copy number on core biopsy FISH of women with breast cancer and HER 2 overexpression, who received combined neoadjuvant therapy at FALP in the period 2017–2020, according to pathologic response at surgery. Both groups were compared by Wilcoxon sign test, resulting in a statistic *W* = 78, *p*-value = 0.004723.

Finally, when comparing the mean value of HER2/centromere ratio (Figure 2a) and HER2 copy number (Figure 2b) described in FISH with the pCR rate, both values were significantly higher in patients with pCR (*W* = 59, *p*-value= 0.003032).

**Figure 3.**
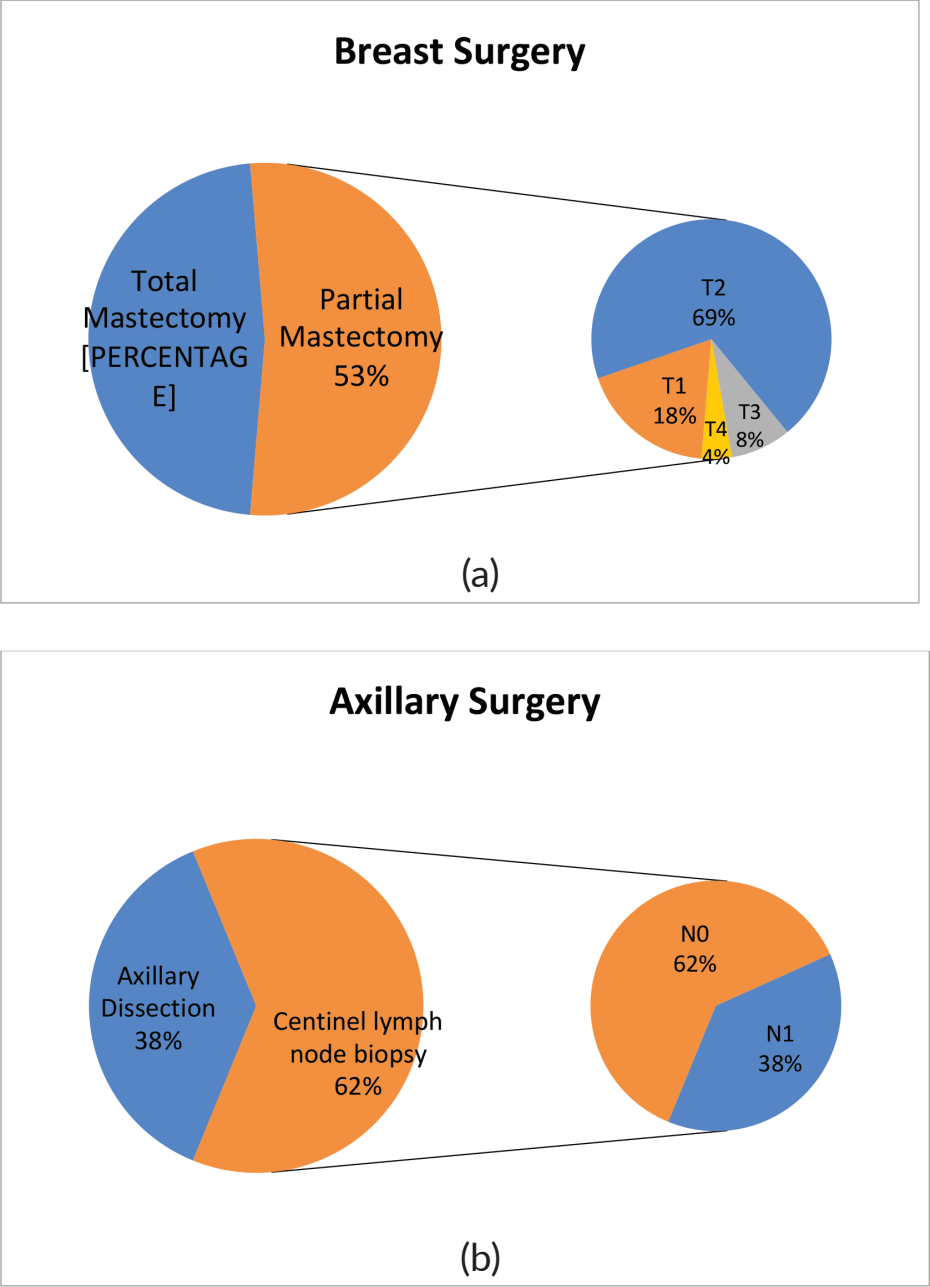
Types of surgery performed after NACT on (a): breast and (b): axilla in patients with breast cancer and HER2 overexpression at FALP between 2017 and 2020.

In 50/94 cases (53%), breast-conserving surgery (partial mastectomy) was performed after combined neoadjuvant therapy. Figure 3a shows the relative importance of local extension strata in the breast (cT) before neoadjuvant therapy in partial mastectomies. In 58/94 (62%) cases, conservative axillary surgery (exclusively sentinel node biopsy) was performed after combined neoadjuvant therapy. Figure 3b shows the relative importance of nodal involvement strata (cN) before neoadjuvant therapy in sentinel node biopsies without axillary dissection.

## Discussion

The 70% of pCR reported in this study with six cycles of TCHP (Carbotaxol + Trastuzumab + Pertuzumab) is somewhat higher than that reported in the TRYPHAENA clinical trial in which case this percentage was 64%. This is the first data obtained in Chile as a result of double anti-HER2 blockade in neoadjuvant treatment with real-world patients. In the case of patients with negative immunohistochemistry for hormone receptors, the rate of pCR was significantly higher (89%), also in agreement with international data and somewhat higher than in the TRYPHAENA study, whose rate of pCR in this group was 84%. It can be inferred that the tumour heterogeneity of HER2/Luminal breast cancer conditions a lower response to neoadjuvant therapies, so much so that the multivariate analysis indicates that the risk of not achieving pCR is 8 times higher in these patients than in those with pure HER2 or hormone receptor-negative tumours. Moreover, having both hormone receptors positive (oestrogen and progesterone), which would speak of an even more luminal tumour, raises the OR of not having pCR to 10 times. This also supports the results found in previous phase 2 studies.

In surgery, the trend over time has been to perform less invasive procedures that have less long-term morbidity, without compromising oncologic safety. The benefit of neoadjuvant treatments in achieving breast conservation is well known, and de-escalation in axillary surgery in patients with positive and negative axilla has been validated [11].

With respect to breast surgery, although it is true that there are conditions that contraindicate breast conservation such as multicentric tumours or tumours with extensive inflammatory and/or local involvement, the tendency is to attempt partial mastectomy. Chemotherapy is a tool that facilitates breast-conserving surgery when there is tumour response, especially in T3 or T2 tumours with poor breast-tumour ratio and that do not allow an adequate cosmetic result. Conservative surgery was performed post NACT in 53% of the cases. Of these, 12% were T3-T4 and 69% were T2. It is necessary to mention that other factors to take into account when deciding on conservative surgery are the patient’s desire, the feasibility of performing subsequent radiotherapy and the presence of mutations in high-risk genes for breast cancer.

Of the 58 cases in which sentinel lymph node biopsy was performed post NACT, 22 were previous N1, so in 38% of patient de-escalation was achieved in axillary surgery. It is important to mention that in our institution, we use a double technique to identify the sentinel lymph node post-NACT and also remove the lymph node or nodes marked with a metal clip, which were positive prior to treatment. This has achieved a false negative rate of less than 10% in N1-2 patients prior to therapy according to international studies, extracting at least three lymph nodes [12, 13].

The results obtained in relation to the association of pCR, HER2/CEN17 ratio elevation and HER2 copy number increase add to the emerging evidence, which differs from the results observed in the first large-scale studies of anti-HER2 therapy. Examples of this are the HERA [14] and NSABP B-31 [15] studies, which found no significant association between these parameters and disease-free survival. More recent studies have shown a possible predictive role for pCR. Arnould *et al* [16] showed in a multivariate analysis that after neoadjuvant therapy patients with high HER 2 amplification (>10 copies/core) had higher pCR than those with low amplification (<6 copies/core) with neoadjuvant therapy with Trastuzumab and taxanes. Similar results were obtained by Choi *et al* [17], with pCR in tumours with a mean HER2/centromere ratio of 7.08 and an average > 17 copies HER2/cell.

In developing this idea, it is possible to hypothesise that a possible cause for not finding a significant relation previously, may be due to the evolution and sophistication of FISH image capture systems, as well as the improvement of processing protocols, currently allowing to obtain high quality images and easy interpretation, unification and greater clarity of the criteria for assessing the results of FISH.

The limitations of our studies include a small sample size, which did not allow us to include more variables in the regression models, and not having the data to calculate the HER2/centromere ratio and the number of HER2 copies in all cases.

## Conclusions

This study provides real-world data on the effect of dual HER-2 blockade in our population of breast cancer patients with HER-2 overexpression. The pCR rates with this treatment strategy are high and comparable to what has been described internationally. To explore the association of pCR with other variables and whether the absence of residual invasive disease post-HER-2 treatment correlates in our centre with an improvement in progression-free survival and overall survival is a matter to be investigated in the future. In our patients as described in other studies, the absence of hormonal receptor expression is a strong predictor of complete pathologic response. We still need more data to show the correlation between pCR, HER2 copy number and HER2/centromere ratio.

The analysis of our results could contribute to the design of public health policies that incorporate high-cost treatments.

## Funding

Financial support for data collection and publication fees was obtained from Roche Laboratory.

## Conflicts of interest

Consulting fees and contracted research for Novartis and Roche.

## Figures and Tables

**Table 1.** Demographic, clinical and histopathological variables in 94 women with breast cancer and HER2 overexpression treated with neoadjuvant at FALP between 2017 and 2020.

Variables	*N* = 94
Median age (range)	53 (32–77)
HER2 (%)	
Pure	36 (38%)
Luminal	58 (62%)
cT (%)	
T1	13 (14%)
T2	55 (59%)
T3	19 (20%)
T4	6 (6%)
Not registered	1 (1%)
cN (%)	
N0	46 (49%)
N1	28 (30%)
N2	12 (13%)
N3	4 (4%)
Not registered	4 (4%)
Chemotherapy scheme (%)	
TCH-P	91 (95%)
Others	5 (5%)
Breast surgery (%)	
Partial or consertvative mastectomy	50 (53%)
Total or radical mastectomy	44 (47%)
Types of pathologic response (%)	
Incomplete	28 (30%)
Complete	66 (70%)

**Table 2.** Demographic, clinical and histopathological variables in 94 women with breast cancer and HER2 overexpression treated with combined neoadjuvant at FALP between 2017 and 2020 according to pathological response post-surgery.

	No pCR *N* = 28	pCR *N* = 66	*p*-value
Age (years)^a^	50 (32–72)	55 (32–77)	0.3595
BMI^a^	28.3 (17.8–51.6)	27.9 (20.4–38.3)	0.9019
Treatment duration (days)^a^	105.5 (71–167)	105 (50–306)	0.9206
TCH-P scheme chemotherapy (%)	96.43% (27)	96.97% (64)	1
Tumour size^a^(mm)	24 (6–90)	26 (3–80)	0.544
Axillary involvement (%)	44.44% (12)	53.85% (35)	0.4943
Stage (%)			
I	44.44% (4)	55.56% (5)	
II	34.48% (20)	65.52% (38)	
III	14.81% (4)	85.19% (23)	<0.05
Molecular subtype and Ki index (%)			
HER2 pure	11.11% (4)	88.89% (32)	
HER 2 Luminal	41.38% (24)	58.62% (34)	<0.05
Ki67 < 20	66.67% (4)	33.33% (2)	
Ki67 > 20	27.27% (24)	72.73% (64)	0.0624

a Continuous variables are presented as median and range

**Table 3.** Effect of clinical and molecular variables on the risk of failing to achieve pCR in a logistic regression model in which hormone receptor expression was dichotomised (HER 2 Luminal versus HER2 Pure) and a logistic regression model in which (ER (+)/PR (+), ER (+), PR (+) and ER (−)/PR (−)) was disaggregated (ER (+)/PR (+), ER (+), PR (+) and ER (−)/PR (−)).

Variables	OR (risk of not achieving pCR)	95% CI
Stage III versus II	0.28	0.067–1,051
Ki67 > 20 versus Ki67 <20	0.19	0.018–1.283
HER2 luminal versus HER2 pure	8.15	2.429–37.728
ER (+)/PR (+) versus ER (**−**)/PR (**−**)	10.42	3.026–47.858
